# Prediction and* In Silico* Identification of Novel B-Cells and T-Cells Epitopes in the S1-Spike Glycoprotein of M41 and CR88 (793/B) Infectious Bronchitis Virus Serotypes for Application in Peptide Vaccines

**DOI:** 10.1155/2016/5484972

**Published:** 2016-09-07

**Authors:** Faruku Bande, Siti Suri Arshad, Mohd Hair Bejo, Saeid Kadkhodaei, Abdul Rahman Omar

**Affiliations:** ^1^Department of Veterinary Pathology and Microbiology, Faculty of Veterinary Medicine, Universiti Putra Malaysia, 43400 Serdang, Selangor Darul Ehsan, Malaysia; ^2^Department of Veterinary Services, Ministry of Animal Health and Fisheries Development, PMB 2109, Usman Faruk Secretariat, Sokoto State, Nigeria; ^3^Laboratory of Vaccine and Immunotherapeutic, Institute of Bioscience, Universiti Putra Malaysia, 43400 Serdang, Selangor Darul Ehsan, Malaysia; ^4^Institute of Tropical Agriculture, Universiti Putra Malaysia, 43400 Serdang, Malaysia

## Abstract

Bioinformatic analysis was used to predict antigenic B-cell and T-cell epitopes within the S1 glycoprotein of M41 and CR88 IBV strains. A conserved linear B-cell epitope peptide, YTSNETTDVTS^175–185^, was identified in M41 IBV strains while three such epitopes types namely, VSNASPNSGGVD^279–290^, HPKCNFRPENI^328–338^, and NETNNAGSVSDCTAGT^54–69^, were predicted in CR88 IBV strains. Analysis of MHCI binding peptides in M41 IBV strains revealed the presence of 15 antigenic peptides out of which 12 were highly conserved in 96–100% of the total M41 strains analysed. Interestingly three of these peptides, GGPITYKVM^208^, WFNSLSVSI^356^, and YLADAGLAI^472^, relatively had high antigenicity index (>1.0). On the other hand, 11 MHCI binding epitope peptides were identified in CR88 IBV strains. Of these, five peptides were found to be highly conserved with a range between 90% and 97%. However, WFNSLSVSL^358^, SYNISAASV^88^, and YNISAASVA^89^ peptides comparably showed high antigenicity scores (>1.0). Combination of antigenic B-cells and T-cells peptides that are conserved across many strains as approach to evoke humoral and CTL immune response will potentially lead to a broad-based vaccine that could reduce the challenges in using live attenuated vaccine technology in the control of IBV infection in poultry.

## 1. Introduction

Infectious bronchitis virus (IBV) is a single stranded, enveloped RNA virus belonging to the family Coronaviridae, order Nidovirales [1]. The virus causes infectious bronchitis (IB), a contagious disease associated with huge economic loses in poultry industry worldwide [2]. Of major concern in the control of IB is the continued emergence of variant IBV strains that differ in terms of their tissue tropism, pathogenicity, and cross protection. Over the years, serological and molecular studies have been carried out extensively to determine the epidemiology of local IBV strains. Remarkably, both classical and variants IBV strains have been reported in different countries [3]. Among the widely identified IBV strains are IBV M41 (classical strain), originally recognized in USA [4, 5], and CR88 (variant strain otherwise known as 793/B or 4/91) which was first reported in Europe [3, 6, 7].

Over the years, control of IBV infection largely depends on vaccination using live attenuated and killed vaccines. However, one of the challenges with live attenuated IB vaccines is that such vaccines are reported to encourage mutation and recombination, thus leading to the emergence of new variant strains. Live attenuated vaccines have also been linked with reversion to virulence, severe disease, and increased mortality rate [8–10]. On the other hand, killed vaccines induce humoral but not cell mediated immune (CMI) response and in most cases require adjuvants as well as repeated boosting especially in laying chickens and breeder flocks. These challenges therefore necessitate the needs for novel broad vaccines for the control of IB in poultry [11]. To achieve this, stimulation of both humoral (B-cell) and cell mediated immune (CMI) responses is considered very essential for any candidate vaccine [12]. Neutralizing antibodies are important in removing freely circulating IB virus, whereas cytotoxic T- lymphocytes (CTL) response is crucial for the control and clearance of virally infected cells. The latter is achieved through MHCI immune surveillance as well as antigen presentations which is the function of MHCI molecule and both have been associated with the epitope within the S1 glycoprotein [13, 14]. While much has been documented on MHC restricted allele in human and mouse models, little information is available on the biological functions of these molecules in poultry [15]. A large binding groove of BF2^*∗*^2101 MHCI molecule identified in B21 chicken line is thought to confer conformational flexibility to the crucial Arg9 residue which allows remodeling of key peptide-binding sites and play a role in the resistance against poultry viral infections [16]. Chicken MHC B–F molecules have been structurally and functionally linked to mammalian MHC class I molecules and involved in antigen presentation to the CD8^+^ T lymphocytes, which is crucial in antiviral immune response [17]. Interestingly, the S1 glycoprotein of IBV (520 aa) contains different immune epitopes responsible for both antibodies and CTL-based immune responses, thus playing major protective role as viral antigenic determinant [18].

Currently, the use of peptide based DNA vaccines represents a novel strategy for addressing challenges associated with the control of viral infections [19, 20]. This technology may employ the use of* in silico* analysis to predict novel B-cells and T-cells immune epitopes for further use in their* in vivo* applications. One of the innovations in using this technology is the ability to incorporate several epitope peptides directed against different viruses and/or multiple virus strains into one single delivery system with the view to induce broad and specific immune response in single administration [11]. To date, only few epitope based peptide vaccines have been developed and evaluated against IB [20, 21]. The objective of this study therefore is to identify novel B-cells and T-cell epitopes within the S1 glycoprotein of M41 and CR88 IBV strains. The antigenicity of the predicted peptides was also evaluated.

## 2. Materials and Methods

### 2.1. Nucleotide Sequence Retrieval

Nucleotide sequences containing complete S1-gene of homologous to M41 and CR88 IBV strains were retrieved from NCBI database and translated into deduced amino acid sequences for further analysis. Consensus amino acid sequences from the two datasets (M41 and CR88) were used to predict B- and T-cells epitope peptides within S1 glycoprotein. The criteria used for the selection of M41 and CR88 were simply based on epidemiological relevance of the two strains in terms of their wide geographic distribution as classical and variant IBV strains, respectively. All sequences were assembled with Geneious® software R7 version [22]. The names and accession numbers of IBV sequences used in this study are presented in Supplementary File 1 in Supplementary Material available online at http://dx.doi.org/10.1155/2016/5484972.

### 2.2. Prediction of B-Cell Epitope

Epitopes associated with B-cells were predicted using the BepiPred epitope prediction server version 1.0 [23]. Similarly, antigenicity index of the predicted epitopes was analysed using VaxiJen v2.0 online antigen prediction tool http://www.ddg-pharmfac.net/vaxijen/VaxiJen/VaxiJen.html [22]. Prediction criteria were set at 75% classifier specificity and 20 epitope lengths with an overlap. The location of the predicted epitope within transmembrane regions was evaluated using TMHMM [24]. Only epitope present at the surface of the membrane was selected and further analysed for antigenicity. For this search, target organism was narrowed to viruses and only scores above 0.4 thresholds were considered as a good antigenic epitope. Further, conservancy analysis was carried out using IEDB tools available http://tools.immuneepitope.org/tools/conservancy/iedb_input [25].

### 2.3. Prediction of T-Cell Epitopes

The T-cell epitope prediction was carried out using ProPred-1 software which covers 47 MHC class-I alleles. Both proteasome and immune-proteasome were set at 5% threshold by MHC class I and only peptides with proteosomal cleavage site at the C terminus were considered [26].

### 2.4. Epitope Conservancy Analysis

Immune epitopes were analysed for conservancy and variability using IEDB conservation analysis tool: http://tools.immuneepitope.org/tools/conservancy/iedb_input [25].

## 3. Results 

### 3.1. Nucleotide Sequence Retrieval

A total of 86 M41 and 72 CR88 IBV isolates were considered in this study. However, to build the consensus sequence, one isolate from each data set was removed due to some unidentified amino acid at one or two positions. The M41 IBV isolates were found to have an average nucleotides sequence length of 1614 (537 aa) while CR88 isolates had an average length of 1653 (553 aa).

### 3.2. B-Cell Epitope Prediction and Conservancy Analysis

Analysis of M41 associated linear B-cell epitopes revealed the presence of six unique B-cells peptides within the S1 glycoprotein. Of these, only YTSNETTDVTS^175–185^ peptide was found to be antigenic (Table 1).

**Figure 1:**
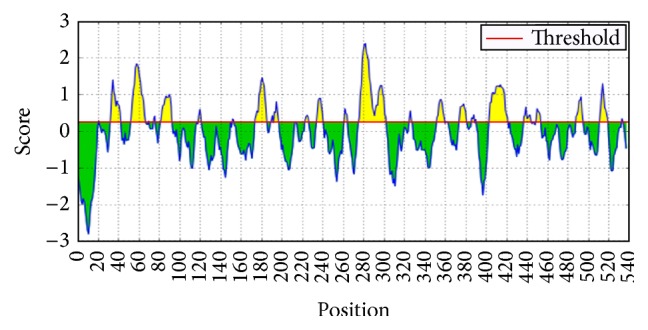
Predicted linear B-cells epitopes and their locations to the surface membrane (yellow) in M41 IBV strains. Note: maximum transmembrane interaction was observed at a threshold of 2.4 within amino acid positions 280 to 290.

Analysis of transmembrane potentials within the M41 IBV S1 gene showed that the predicted linear B-cell epitopes interact with the outside surface membrane especially with maximum threshold occurring at amino acid positions 280 to 290 (Figure 1).

Interestingly, conservancy analysis showed that YTSNETTDVTS^175–185^ peptide is common in 82.35% of the M41 strains. On the other hand, a total of six antigenic epitope peptides were predicted in CR88 IBV strains; however, three of these peptides, NETNNAGSVSDCTAGT^54–69^, VSNASPNSGGVD^279–290^, and HPKCNFRPENI^328–338^ were demonstrated to be antigenic (Table 2). Further, conservancy analysis showed that the three CR88 associated antigenic B-cell peptides had 39.44%, 84.5%, and 43.66% conservancy rates, respectively.

**Figure 2:**
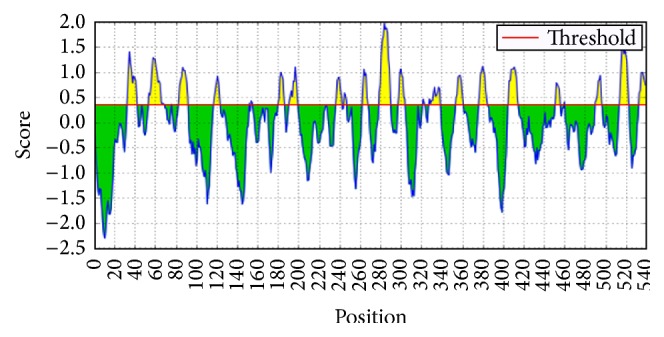
Linear B-cells epitope and their interaction with the surface membrane (yellow) in CR88 IBV strains. Note: the high east transmembrane interaction was predicted at the threshold of about 1.9 within the amino acid positions 280–290.

As with the M41 IBV strains, most of these epitopes are shown to interact with the surface at varying threshold (yellow) as depicted in Figure 2.

### 3.3. Prediction of T-Cell Epitope and Conservancy Analysis

#### 3.3.1. Prediction of MHC-I Binding Epitopes

In M41 IBV strains, a total of 21 MHCI peptides were predicted at different amino acid positions; however, 15 of these peptides were found to be antigenic at various conservancy rates (98.82–100%). Three epitope peptides, GGPITYKVM^208^, WFNSLSVSI^356^, and YLADAGLAI^472^ relatively have high antigenicity index (>1.0) as compared to other peptides (Table 3).

In the case of CR88 IBV strains, 18 MHCI epitope binding peptides were predicted. Of these, 11 peptides were found to be antigenic with most of the epitopes having conservancy rate ranging from 52.11% to 94.37% except peptide occurring at position S69 which is conserved in only 8.45%. Interestingly, WFNSLSVSL^358^, SYNISAASV^88^, and YNISAASVA^89^ demonstrated high antigenicity index compared to other predicted peptides (Table 4).

## 4. Discussion

The use of bioinformatic and/or* in silico* analyses to understand infectious diseases as well as to predict novel vaccine candidate has been recently extended to poultry [27, 28]. Most of the epitopes responsible for virus neutralization have been mapped to be located within the first and third quarters of the linear S1 glycoprotein [13, 16]. The present study identified novel B-cells and T-cells epitopes presence in the S1 glycoprotein of M41 and CR88 IBV strains. Predicted antigenic B-cell epitopes were found to be highly conserved and demonstrated strong transmembrane potentials which probably predicts regions of virus-cell interaction. Linear B-cells epitopes located within the S1 region have been reported to play a role in virus neutralization. Using a phage display library, Zou et al. [29] identified two neutralizing linear B-cells epitopes within the S1 glycoprotein.

Recent studies have shown that chicken MHCI genes are categorized into MHCI associated genes (B-F) and MHCII (B-L) associated B-G genes, probably found only in poultry. Remarkably, this study has identified 21 and 18 MHCI binding peptides located within the S1 glycoproteins of M41 and CR88 IBV strains, respectively. Chickens with MHC homozygous B12 and B19 are reported to be more susceptible to infection with IBV-Grey strains as compared with B2/B2 and B5/B5 haplotype which often resist infection [30]. Recently, Tan et al. [21] used BF2^*∗*^4, BF2^*∗*^12, BF2^*∗*^15, and BF2^*∗*^19 chicken MHCI haplotypes to predict 21 CTL-peptide candidates in Massachusetts and Australian T strains. The study revealed that the constructed poly-CTL-epitope DNA vaccine was capable of inducing protection in 90% of the vaccinated chicken following challenge with SH1208 IBV strain. In a different study, Tian et al., 2008 [31] have identified seven T- and B-cells epitopes within the IBV S1 and S2 and NP proteins chimeric DNA vaccine derived from these epitope was found to be associated with 80% protection rate. Interestingly, the regions through which B-cells and T-cells epitope were predicted in this study correspond to the IBV receptor binding domain (RBD) which play a role in sufficient binding as well as entry of IBV viruses [32]. Similarly, epitopes peptides found within RBD have been reported in different study to induce neutralizing antibody response, for example, against human coronavirus and mouse hepatitis virus [33, 34].

## 5. Conclusion

This study predicted novel antigenic B-cells and T-cell epitopes within the S1 glycoprotein of M41 and CR88 IBV strains. The characteristic antigenicity index as well as epitope conservancy rates demonstrates potentials of the identified epitope peptides as polyvalent synthetic or DNA-based peptide vaccine for application in the control of IBV infection. The use of such vaccines will likely reduce the challenges associated with live attenuated vaccines and allow broad coverage of the target IBV strains.

## Supplementary Material

M41 and CR88-like sequences retrieved from GenBank and used for bioinformatic and Epitope prediction analysis.

## Figures and Tables

**Table 1:** Predicted antigenic B-cells linear epitopes within the S1 glycoprotein sequence of M41 IBV strains.

Position	Peptides	Length	Antigenicity
S33–44	AFRPGPGWHL	10	0.1545
S52–66	SSESNNAGSSSGCT	14	0.0842
S83–92	MTAPSSGMAW	10	0.0728
*S175*–*185*	*YTSNETTDVTS*	*11*	*0.6727*
S403–421	KSGGSRIQTATEPPVITQH	19	0.2319

Peptides in italic represent antigenic epitope considered.

**Table 2:** Predicted antigenic B-cells linear epitopes found in the S1 glycoprotein sequence of CR88 IBV strains.

Epitope position	Peptides	Length	Antigenicity
S33–44	AFRPGPGWHL	10	0.1545
*S54*–*69*	*NETNNAGSVSDCTAGT*	*16*	*0.4237*
S83–92	MTVPPNGMSW	10	0.0306
*S279*–*290*	*VSNASPNSGGVD*	*12*	*0.7396*
*S328*–*338*	*HPKCNFRPENI*	*11*	*1.9437*
S405–414	KSDGSRIQTR	10	0.3178

Peptides in italic represent antigenic epitope.

**Table 3:** Prediction of MHCI binding epitopes in the S1 glycoprotein of M41 IBV strains and their characteristic antigenicity scores.

Epitope position	Peptide sequence	Conservancy %	Antigenicity scores
18	VTPLLLVTL	98.82	0.8935
19	TPLLLVTLL	9882	0.7935
176	VNNLTSVYL	98.82	0.6733
192	SNETTDVTS	82.35	0.6575
208	GGPITYKVM	100	1.3364
214	KVMREVKAL	80.00	0.4479
356	WFNSLSVSI	98.82	1.1016
387	AYSYGGPSL	49.41	0.9013
462	NVTDSAVSY	100	0.6936
465	DSAVSYNYL	100	0.9786
467	AVSYNYLAD	100	0.5294
470	YNYLADAGL	96.47	0.5650
471	NYLADAGLA	96.47	0.9234
472	YLADAGLAI	96.47	1.0681
513	FVVSGGKLV	98.82	0.6146

Note: only epitopes with 0.4 and above scores are presented.

**Table 4:** MHCI associated antigenic peptide predicted on the S1 glycoprotein of CR88 IBV strains.

Epitope position	Peptide sequence	Conservancy %	Antigenicity
S18	GKPLLLVTL	69.01	0.9669
S69	NETNNAGSV	8.45	0.5716
S88	SYNISAASV	52.11	1.1186
S89	YNISAASVA	52.11	1.1067
S216	KVMKEVKAL	90.14	0.432
S358	WFNSLSVSL	80.28	1.1441
S467	EATANYSYL	94.37	0.8157
S472	YSYLADGGL	94.37	0.512
S473	SYLADGGLA	94.37	0.8439
S474	YLADGGLAI	97.18	0.9277
S479	GLAILDTSG	94.37	0.4147
