# Supplementary material for: Prediction and In Silico Identification of Novel B-Cells and T-Cells Epitopes in the S1-Spike Glycoprotein of M41 and CR88 (793/B) Infectious Bronchitis Virus Serotypes for Application in Peptide Vaccines
Source: Adv Bioinformatics. 2016 Sep 7;2016:5484972. doi: 10.1155/2016/5484972 (PMC5030393; doi:10.1155/2016/5484972)
Supplement: Supplementary file 1 — M41 and CR88-like sequences retrieved from GenBank and used for bioinformatic and Epitope prediction analysis. [file 5484972.f1.docx]

Supplementary material 1: List of CR88 and M41 IBV strains used in epitope prediction analysis

| CR88-LIKE ISOLATES | | | MASSACHUSETTS-LIKE ISOLATES | | |
| --- | --- | --- | --- | --- | --- |
| Isolate | Accession number | Country of isolation | Isolate | Accession number | Country of isolation |
| 4_91_UK | JN192154 | UNITED KINGDOM | 1489/99 | JN022543 | SWEDEN |
| 4_91_attenuated | AF093793 | UNITED KINGDOM | 25SDZC-01 | KC577371 | CHINA |
| 4_91_pathogenic | AF093794 | UNITED KINGDOM | 261/95 | JN022537 | SWEDEN |
| 4_91_vaccine | KF377577 | UNITED KINGDOM | 31JL-97I | KC577375 | CHINA |
| ck_CH_LHLJ_111246 | JQ739319 | CHINA | 33JS-95III | KC577377 | CHINA |
| ck_CH_LSD_110410 | JQ739353 | CHINA | IB900419 | JN022554 | SWEDEN |
| ck_CH_LSD_110851 | JQ739372 | CHINA | A889 | JN022555] | SWEDEN |
| ck_CH_LSD_110857 | JQ739375 | CHINA | AH07091 | FJ829873 | CHINA |
| CR88121 | JN542567 | FRANCE | AH07101 | FJ829874 | CHINA |
| CR88_UPM2013 | KM067900 | MALAYSIA | AH240 | FJ829880 | CHINA |
| FR9404794 | AJ618987 | UNITED KINGDOM | Beaudette-P36 | DQ001342 | SINGAPORE |
| FR_AJ618985 | AJ618985 | UNITED KINGDOM | Beaudette-Vero_adapted | AY692454 | USA |
| FRCR8806188 | AJ618986 | UNITED KINGDOM | Beaudette | M95169 | UK |
| Spain_97_307 | DQ064805 | SPAIN | Beaudette_42 | DQ830981 | USA |
| Spain_92_35 | DQ386091 | SPAIN | Beaudette_CK | AJ311317 | UK |
| GXNN7 | FJ907239 | CHINA | Beaudette_US(Vero_cell_adapted) | AJ311362 | UK |
| IBV005 | KF809770 | INDIA | Beaudette_variant1 | DQ001334 | SINGAPORE |
| IBV014 | KF809771 | INDIA | ck/CH/LDL/110931 | JQ739237 | CHINA |
| IBV025 | KF809774 | INDIA | ck/CH/LDL/110931 | KJ425485 | CHINA |
| IBV136 | KF809775 | INDIA | ck/CH/LHB/131142 | KJ425501 | CHINA |
| IBV151 | KF809776 | INDIA | ck/CH/LHLJ/091205 | HM194672 | CHINA |
| IBV208 | KF809777 | INDIA | ck/CH/LHLJ/091205 | KJ425504 | CHINA |
| IBV267 | KF809782 | INDIA | ck/CH/LHLJ/100902 | JF330855 | USA |
| IBV572 | KF809797 | INDIA | ck/CH/LHLJ/100902 | JF828980 | CHINA |
| IBV573 | KF809798 | INDIA | ck/CH/LJL/110654 | JQ739327 | CHINA |
| IBV586 | KF809799 | INDIA | ck/CH/LSD/1112150 | KJ435286 | CHINA |
| IBVIR3654VM | AY544776 | IRAN | Egypt/F/03 | DQ487085 | EGYPT |
| IR_14_07 | HQ842712 | IRAN | GX1-98 | AY319302 | CHINA |
| IR_19_08 | HQ842714 | IRAN | H120 | KF188436 | INDIA |
| IR_491_08 | HQ842715 | IRAN | H52[EU817497]CHINA | EU817497 | CHINA |
| IR_512_99 | HQ842707 | IRAN | IBV/Brasil/PM3/1989 | GU393341 | BRAZIL |
| IR_525_99 | HQ842708 | IRAN | IBV431 | KF809792 | INDIA |
| IR_573_98 | HQ842706 | IRAN | IBV628 | KF809803 | INDIA |
| IR_803_03 | HQ842711 | IRAN | IBV_N | FJ829881 | CHINA |
| IR1061PH | AY544778 | IRAN | JD071201 | FJ829878 | CHINA |
| IR1062GA | AY544777 | IRAN | JH051 | FJ829875 | CHINA |
| IS_1366 | EU350550 | ISRAEL | JH06011 | FJ829877 | CHINA |
| JP_Iwate_2005 | AB363959.2 | JAPAN | JH06111 | FJ829876 | CHINA |
| JP_Saitama_2006 | AB465727 | JAPAN | Jin-13 | GU455379 | CHINA |
| JP_Wakayama_2003 | AB363949.2 | JAPAN | JL/97/01 | AF258780 | CHINA |
| JP_Wakayama2_2004 | AB363951.2 | JAPAN | JS/95/03 | AF208239 | CHINA |
| MoroccanG_83 | EU914938 | MOROCCO | K110/06 | FJ807921 | S.KOREA |
| Sichuan06 | GQ844991 | CHINA | K446-01 | AY257063 | S.KOREA |
| Spain_00_336 | DQ386098 | SPAIN | L423 | JQ964062 | USA |
| Spain_92_185 | DQ386092 | SPAIN | L455 | JQ964063 | USA |
| Spain_92_51 | DQ064801 | SPAIN | L569 | JQ964065 | USA |
| Spain_95_193 | DQ386093 | SPAIN | L748 | JQ964068 | USA |
| Spain_95_194 | DQ064802 | SPAIN | L74 | JQ964060 | USA |
| Spain_96_312 | DQ064803 | SPAIN | L806 | JQ964069 | USA |
| Spain_96_330 | DQ386094 | SPAIN | L969 | JQ964072 | USA |
| Spain_98_315 | DQ386095 | SPAIN | M26 | F391155 | USA |
| Spain_98_328 | DQ386096 | SPAIN | M28 | F391156 | USA |
| 99_316 | DQ064809 | SPAIN | M41-UPM-2013(ATCC) | KM067901 | MALAYSIA |
| Spain_99_319 | DQ064810 | SPAIN | M41_USA | AY561711 | USA |
| Spain_99_325 | DQ064811 | SPAIN | M41_INDIA | GQ219712 | INDIA |
| Spain_99_326 | DQ064812 | SPAIN | Mass/B13dpvcontact | EU283081 | USA |
| Spain_99_327 | DQ386097 | SPAIN | Mass/B3dpvvaccinated- | EU283078 | USA |
| strain_A | AF151953 | NEW ZEALAND | Mass/B6dpvcontact | EU283080 | USA |
| strain_B | AF151954 | NEW ZEALAND | Mass/B9dpvvaccinated | EU283079 | USA |
| strain_C | AF151955 | NEW ZEALAND | Mass/Bvial1 | EU283076 | USA |
| Strain_D | AF151956 | NEW ZEALAND | Mass/Bvial2 | EU283077 | USA |
| TaO3 | AY837465 | CHINA | Mass/C_reisolated | EU283084 | USA |
| Taian03 | GQ844992 | CHINA | Mass/Cvial1 | EU283082 | USA |
| UK_2_91 | Z83976 | UNITED KINGDOM | Mass/Cvial2 | EU283083 | USA |
| UK_5_91 | Z83978 | UNITED KINGDOM | Mass41_1965 | FJ904720 | USA |
| UK_7/91 | Z83975 | UNITED KINGDOM | Mass41_1972 | FJ904721 | USA |
| UK_7/93 | Z83979 | UNITED KINGDOM | Mass41_1979 | FJ904722 | USA |
| UK1233/95 | AJ618984 | UNITED KINGDOM | Mass41_1985 | FJ904723 | USA |
| VAR233A | JQ946056 | ISRAEL | Mass41_Vaccine | GQ504725 | USA |
| VAR-1 | AF093795 | ISRAEL | Mass_vaccine | EU359657 | USA |
| IBV018 | KF809772 | INDIA | Massachusetts_1941 | GQ504724 | USA |
| IBV022 | KF809773 | INDIA | Massachusetts_41 | DQ664534 | USA |
|  |  |  | Massachusetts_41 | DQ830980 | USA |
|  |  |  | Massachusetts_41 | DQ834384 | USA |
|  |  |  | MassD/Cvial2 | EU283086 | USA |
|  |  |  | NV_M41 | AY561712 | USA |
|  |  |  | Peafowl/GD/KQ6/2003 | AY641576 | CHINA |
|  |  |  | SC021201 | FJ829883 | CHINA |
|  |  |  | SDA | AY043313 | CHINA |
|  |  |  | Spain/96/334 | DQ064804 | SPAIN |
|  |  |  | Strain/41-VACCINE | M21883 | EUROPE |
|  |  |  | THA280252 | GQ885134 | THAILAND |
|  |  |  | THA290252 | GQ885135 | THAILAND |
|  |  |  | UFMG/200 | JX182789 | BRAZIL |
|  |  |  | UFMG/PM3 | JX182771 | BRAZIL |
|  |  |  | UFMG/PM4 | JX182770 | BRAZIL |
